# High VEGF Concentrations Accelerate Human Trabecular Meshwork Fibrosis in a TAZ-Dependent Manner

**DOI:** 10.3390/ijms24119625

**Published:** 2023-06-01

**Authors:** Mi Sun Sung, So Young Kim, Gwang Hyeon Eom, Sang Woo Park

**Affiliations:** 1 Department of Ophthalmology, Chonnam National University Medical School and Hospital, Gwangju 61469, Republic of Korea; sms84831@hanmail.net (M.S.S.); syou8246@naver.com (S.Y.K.); 2 Department of Pharmacology, Chonnam National University Medical School, Hwasun 58128, Republic of Korea; eomgh@chonnam.ac.kr

**Keywords:** VEGF, human trabecular meshwork, fibrosis, TAZ

## Abstract

We aimed to investigate the effects of different concentrations of vascular endothelial growth factor (VEGF) on the extracellular matrix (ECM) and fibrotic proteins in human trabecular meshwork (TM) cells. We also explored how the Yes-associated protein (YAP)/transcriptional co-activator with PDZ-binding motif (TAZ) signaling pathway modulates VEGF-induced fibrosis. We determined cross-linked actin network (CLAN) formation using TM cells. Changes in fibrotic and ECM protein expression were determined. High VEGF concentrations (10 and 30 ng/mL) increased TAZ and decreased p-TAZ/TAZ expression in TM cells. Western blotting and real-time PCR revealed no YAP expression changes. Fibrotic and ECM protein expression decreased at low VEGF concentrations (1 and 10 ρg/mL) and significantly increased at high VEGF concentrations (10 and 30 ng/mL). CLAN formation increased in TM cells treated with high VEGF concentrations. Moreover, TAZ inhibition by verteporfin (1 μM) rescued TM cells from high-VEGF-concentration-induced fibrosis. Low VEGF concentrations reduced fibrotic changes, whereas high VEGF concentrations accelerated fibrosis and CLAN formations in TM cells in a TAZ-dependent manner. These findings reflect the dose-dependent influences of VEGF on TM cells. Moreover, TAZ inhibition might be a therapeutic target for VEGF-induced TM dysfunction.

## 1. Introduction

Glaucoma is a progressive, irreversible damage of retinal ganglion cells that results in a characteristic optic disc change and a corresponding visual field defect [1]. It is the second leading cause of blindness worldwide, affecting over 60 million people [2]. Among the several causative factors, elevated intraocular pressure (IOP) is the major risk factor for glaucoma development and progression [3]. The balance between the production and outflow of the aqueous humor is essential for maintaining normal physiologic IOP levels. When the aqueous humor drainage resistance increases, the IOP is elevated. In particular, the conventional route via the trabecular meshwork (TM) is the major site of aqueous outflow.

Endothelial-like TM cells are located along the series of fenestrated beams and sheets of TM. TM cells are mechanoresponsive and have contractile properties. Moreover, they secrete a number of extracellular matrix (ECM) proteins and degradation enzymes to support the continuous remodeling of ECM. Through these capacities to perform a variety of functions, they dynamically regulate the tissue resistance to aqueous outflow. IOP homeostasis depends largely on the TM properties. A number of changes in the anatomical and functional characteristics of TM cells have been reported in glaucomatous eyes, and resistance produced in the TM is mainly responsible for the IOP elevation in glaucoma [4,5]. For these reasons, the TM is a subject of intense research in the glaucoma field.

Vascular endothelial growth factor (VEGF) is a crucial therapeutic target in ophthalmology. Anti-VEGF discovery has revolutionized the management of age-related macular degeneration, diabetic macular edema, and retinal vein occlusion. VEGF causes pathological disease changes; nonetheless, it also plays a crucial role in maintaining outflow facilities in physiological conditions. As a potent regulator of endothelial permeability, VEGF is known to regulate the permeability of Schlemm’s canal (SC) [6,7,8,9]. Recent research has proposed that VEGF is secreted by human TM cells in response to IOP-dependent mechanical forces. Such stretch-induced VEGF production may provide a mechanosensitive feedback signal to modulate the outflow facility for IOP homeostasis [8]. However, the mechanisms by which VEGF regulates the outflow facility remain not fully understood. Considering the widespread use of anti-VEGF therapy in ophthalmology, even though these agents act differently [10], understanding its precise role in TM and the molecular mechanism of interaction between them might be clinically important.

Yes-associated protein (YAP) and its transcriptional co-activator with PDZ-binding motif (TAZ)—the Hippo pathway effectors—have been identified as key mechanotransducers that sense mechanical stimuli and relay the signals to control transcriptional programs for cell proliferation and differentiation [11,12,13]. Under mechanical stress, YAP/TAZ are translocated to the nucleus and subsequently activated by binding to TEA/TEF-domain transcription factors (TEAD) [11,12,13,14]. Structural TM changes observed in glaucoma are influenced by YAP/TAZ. Studies have also observed that certain glaucoma-related molecules that influence outflow function, such as dexamethasone, lysophosphatidic acid, and interleukin 6, mediate YAP/TAZ expression in human TM cells [15,16,17,18,19,20,21,22,23]. In addition, Wang et al. [24] recently proposed that YAP/TAZ play central roles in regulating VEGF signaling. Here, we hypothesized that VEGF’s role as a regulator of the TM outflow facility might be related to YAP/TAZ activity. We investigated whether VEGF affects ECM and fibrotic protein expression in human TM cells to test our hypothesis. Next, we explored how the YAP/TAZ signaling pathway modulates VEGF-induced fibrosis in human TM cells.

## 2. Results

### 2.1. Effects of VEGF on the Viability of Human TM Cells

**Figure 1:**
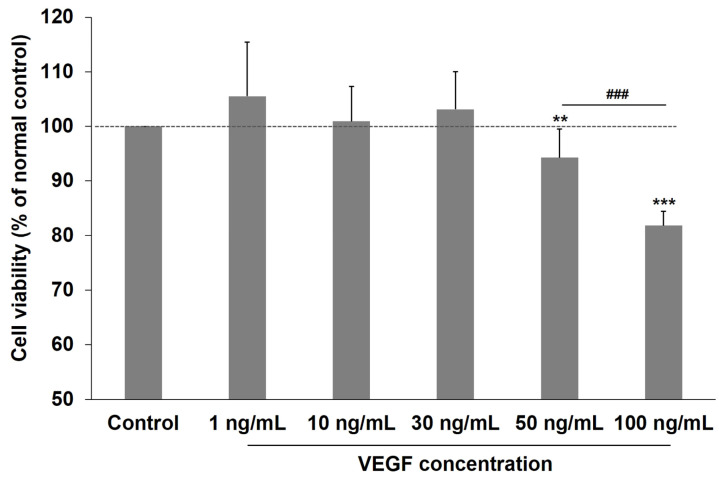
CCK-8 assay of human TM cells after exposure to different VEGF concentrations. The CCK-8 assay revealed that incubating human TM cells with 1–30 ng/mL VEGF does not significantly influence cell viability. However, the viability decreased significantly at 50 and 100 ng/mL VEGF in a dose-dependent manner. Asterisks (*) are used to show comparisons between the group of interest versus control cells; ** *p* < 0.01, *** *p* < 0.001. Hash symbol (#) denotes the results between the cells exposed to 50 and 100 ng/mL VEGF; ^###^ *p* < 0.001. Significance was determined using one-way ANOVA followed by Turkey’s multiple comparisons post-hoc test.

To ascertain the appropriate VEGF exposure dosage, we evaluated the effects of various VEGF concentrations on human TM cell viability. Figure 1 reveals that VEGF exposure (0–30 ng/mL) did not significantly affect human TM cell viability. However, viability decreased significantly at 50 ng/mL VEGF compared with the unexposed control group (*p* < 0.01). Furthermore, cell viability further decreased when VEGF concentration increased to 100 ng/mL (both *p* < 0.001; control vs. 100 ng/mL VEGF and 50 ng/mL VEGF vs. 100 ng/mL VEGF). Therefore, VEGF at 30 ng/mL was the highest concentration used for the experiments.

### 2.2. High VEGF Concentrations Increase TAZ Activity in Human TM Cells

**Figure 2:**
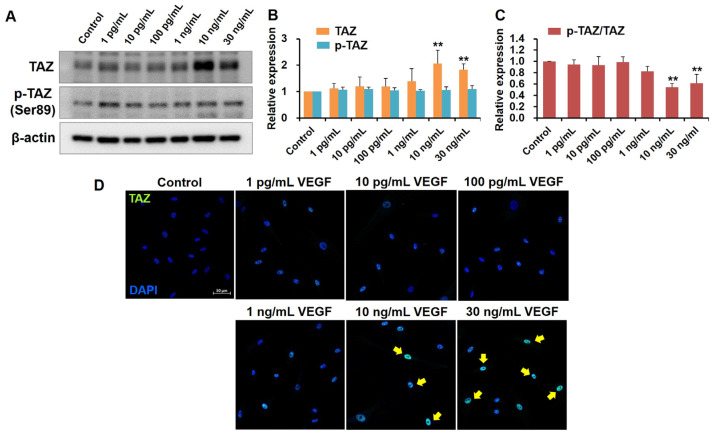
High VEGF concentrations promote TAZ expression and nuclear localization in human TM cells. (**A**) Western blot analysis of TAZ protein and its phosphorylation in human TM cells at various VEGF concentrations. β-actin was the internal control. (**B**) Expression of TAZ and p-TAZ/TAZ was significantly upregulated at 10 and 30 ng/mL VEGF, whereas that of p-TAZ was not changed by VEGF exposure. (**C**) Quantification of p-TAZ abundance relative to TAZ. (**D**) Representative image demonstrating the subcellular localization of TAZ (*green*) and nuclei (*blue*) in human TM cells across the various VEGF concentrations. The *Yellow arrows* indicate TAZ localization in the nucleus. Asterisks (*) are used to show comparisons between the group of interest versus control cells; ** *p* < 0.01. Significance was determined using one-way ANOVA followed by Turkey’s multiple comparisons post-hoc test.

To determine whether VEGF’s role as an outflow facility regulator in TM is related to YAP/TAZ activity, we first studied VEGF’s effects on YAP and TAZ expression in human TM cells. Human TM cells were incubated with various VEGF concentrations. TAZ expression did not significantly differ in cultured human TM cells subjected to low VEGF concentrations (1 ρg/mL, 10 ρg/mL, 100 ρg/mL, and 1 ng/mL) compared with the control cells. However, TAZ expression was significantly upregulated at 10 and 30 ng/mL of VEGF (both *p* < 0.01, compared with the control cells; Figure 2A,B). TAZ phosphorylation at Ser 89, leading to TAZ cytoplasmic sequestration, remained relatively constant regardless of VEGF concentration. Accordingly, phosphorylated TAZ (p-TAZ) abundance relative to that of total TAZ in human TM cells decreased at high VEGF concentrations (Figure 2C). Immunofluorescence staining for TAZ revealed that the human TM cells subjected to high VEGF concentrations (10 and 30 ng/mL) exhibited increased nuclear localization than the control cells, indicating TAZ activation (Figure 2D).

### 2.3. YAP Expression Unaffected by VEGF Exposure in Human TM Cells

**Figure 3:**
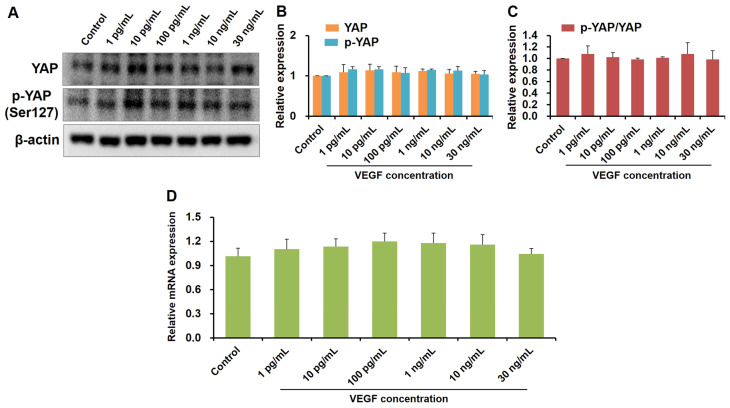
YAP expression was unaffected by VEGF exposure in human TM cells. (**A**) Western blot analysis of YAP protein and its phosphorylation in human TM cells at various VEGF concentrations. Β-actin was the internal control. No significant change in YAP protein expression was observed compared with the control cells. (**B**) YAP and p-YAP expression. (**C**) p-YAP abundance quantification, relative to YAP. (**D**) YAP mRNA expression. Consistent with the results in protein levels, YAP mRNA expression did not significantly differ across the various VEGF concentration groups.

YAP and TAZ are generally thought to function similarly in response to mechanical and biochemical signals. However, their distinct effects have been reported and several studies highlight their differential roles [25]. Interestingly, we observed that YAP expression in response to VEGF exposure differed from that of TAZ in this study. The protein expression of YAP and phosphorylated YAP (p-YAP) was unaltered by VEGF exposure in human TM cells. Consistent with the protein levels, mRNA expression of YAP did not significantly differ across the various VEGF concentration groups (Figure 3).

### 2.4. Dose-Dependent Influences of VEGF on Fibrotic Protein Expression in Human TM Cells

**Figure 4:**
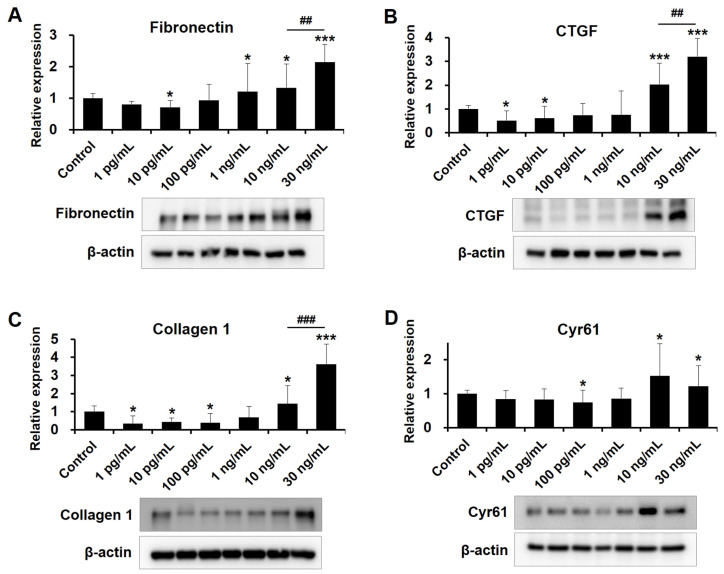
VEGF affects the expression level of fibrotic proteins in human TM cells. Levels of fibronectin, CTGF, collagen 1, and Cyr61 were determined via Western blotting. Β-actin was the internal control. (**A**) Immunoblot of fibronectin. (**B**) Immunoblot of CTGF. (**C**) Immunoblot of collagen 1. (**D**) Immunoblot of Cyr61. Asterisks (*) are used to show comparisons between the group of interest versus control cells; * *p* < 0.05, *** *p* < 0.001. Hash symbol (#) denotes the results between the cells exposed to 10 and 30 ng/mL VEGF; ^##^ *p* < 0.01, ^###^ *p* < 0.001. Significance was determined using one-way ANOVA followed by Turkey’s multiple comparisons post-hoc test.

Complex ECM proteins surround human TM cells, and increased deposition of ECM proteins and transforming growth factor-β (TGF-β)-induced fibrosis increase aqueous humor outflow resistance and IOP elevation [26]. Therefore, we explored the effects of various VEGF concentrations on ECM protein expression in human TM cells. Overall, as revealed by Western blotting in Figure 4, low-concentration VEGF suppressed the expression of TGF-β2-induced fibrotic proteins, such as fibronectin, CTGF, collagen 1, and Cyr61 in human TM cells. Specifically, fibronectin was significantly decreased at 10 ρg/mL VEGF and CTGF expression was significantly decreased at 1 and 10 ρg/mL VEGF. Collagen 1 was significantly decreased at 1, 10, and 100 ρg/mL VEGF. Cyr61 significantly decreased at 100 ρg/mL VEGF, whereas exposure to high VEGF concentrations (10 and 30 ng/mL) upregulated fibrotic proteins in human TM cells. In addition, fibronectin, CTGF, and collagen 1 expression levels were significantly higher at 30 ng/mL VEGF than at 10 ng/mL. These results suggest a dose-dependent influence of VEGF on the human TM cells.

### 2.5. Exposure to High-Concentration VEGF Promotes CLAN Formation in Human TM Cells

**Figure 5:**
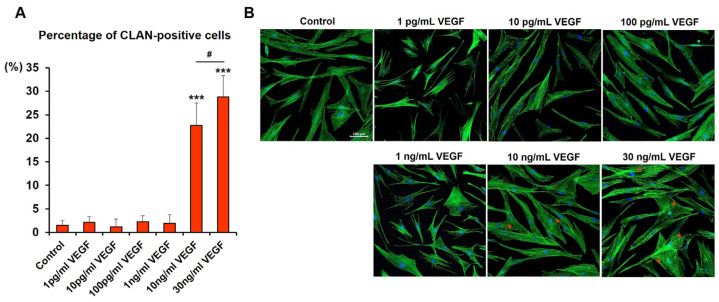
High VEGF concentrations promote CLAN formation in human TM cells. (**A**) Percentage of CLAN-positive cells is presented as the mean ± SD. Cells exposed to 10 ng/mL (22.72% ± 4.80%; *p* < 0.001, compared with the control cells) and 30 ng/mL VEGF (28.84% ± 4.52%; *p* < 0.001, compared with the control cells; *p* = 0.02, compared with cells exposed to 10 ng/mL VEGF) had a higher percentage of CLAN-positive cells than unexposed cells. Asterisks (*) are used to show comparisons between the group of interest versus control cells; *** *p* < 0.001. Hash symbol (#) denotes the results between the cells exposed to 10 and 30 ng/mL VEGF; ^#^ *p* < 0.05. Significance was determined using one-way ANOVA followed by Turkey’s multiple comparisons post-hoc test. (**B**) Representative images of actin stress fibers. Red asterisks represent CLANs. 3.6. Verteporfin reversed high-concentration VEGF-induced fibrosis and actin cytoskeleton changes in human TM cells.

Changes in the actin cytoskeleton, especially CLAN formation, contribute to the increased aqueous humor outflow resistance in patients with glaucoma [27,28]. CLANs are three-dimensional, geodesic dome-like structures formed primarily around the nucleus thought to increase the actin filament’s stiffness, leading to altered TM contractility [27,28,29]. Compared with the control cells, human TM cells cultured with high VEGF concentrations (10 and 30 ng/mL) exhibited significant increases in CLAN formation in a dose-dependent manner (Figure 5). In unexposed human TM cells, the percentage of CLAN-positive cells was 1.52% ± 0.97%. In contrast, the percentages of CLAN-positive cells in cultures exposed to 10 and 30 ng/mL VEGF were 22.72% ± 4.80% and 28.84% ± 4.52%, respectively (both *p* < 0.001, compared with the control cells; *p* = 0.02, compared with the 10 and 30 ng/mL VEGF groups).

**Figure 6:**
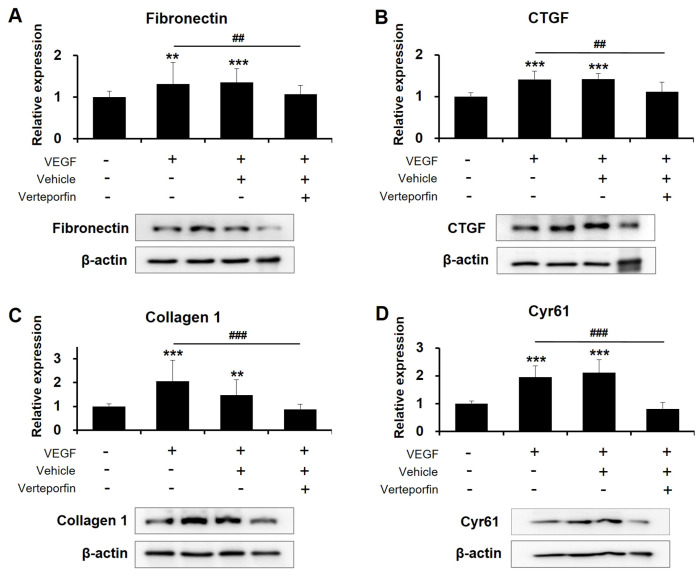
TAZ activity inhibition via verteporfin attenuates VEGF-induced fibrosis in human TM cells. (**A**–**D**) Verteporfin inhibited high-VEGF-concentration-induced increases in fibrosis in human TM cells similarly. Expression levels of fibronectin (**A**), CTGF (**B**), collagen 1 (**C**), and Cyr61 (**D**) were examined using Western blotting. Asterisks (*) are used to show comparisons between the group of interest versus control cells; ** *p* < 0.01, *** *p* < 0.001. Hash symbol (#) denotes the results between the cells exposed to 30 ng/mL VEGF only and the cells subsequently treated with verteporfin after VEGF exposure; ^##^ *p* < 0.01, ^###^ *p* < 0.001. Significance was determined using one-way ANOVA followed by Turkey’s multiple comparisons post-hoc test.

**Figure 7:**
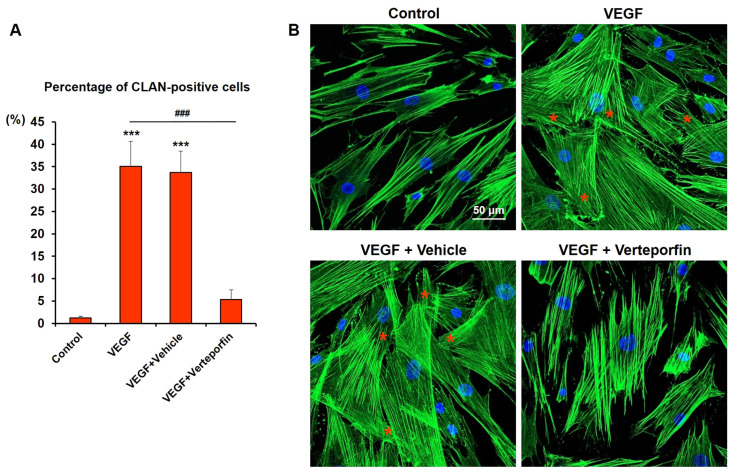
Verteporfin resolved the high-VEGF-concentration-induced CLAN structure in human TM cells. (**A**) Percentage of CLAN-positive cells is presented as the mean ± SD. Cells exposed to 30 ng/mL VEGF demonstrated a markedly increased CLAN formation compared with the unexposed control cells (1.23% ± 0.45% vs. 35.11% ± 5.60%, *p* < 0.001). Treatment with verteporfin significantly attenuated CLAN structures (5.37% ± 2.24%, *p* < 0.001 compared with the untreated VEGF-exposed cells). Asterisks (*) are used to show comparisons between the group of interest versus control cells; *** *p* < 0.001. Hash symbol (#) denotes the results between the cells exposed to 30 ng/mL VEGF only and the cells subsequently treated with verteporfin after VEGF exposure; ^###^ *p* < 0.001. Significance was determined using one-way ANOVA followed by Turkey’s multiple comparisons post-hoc test. (**B**) Representative images of the actin stress fibers. Red asterisks represent CLANs.

To determine whether the effects of high VEGF concentrations on the human TM cells are associated with TAZ activity, we examined the effects of a potent YAP/TAZ inhibitor—verteporfin. Studies have revealed that verteporfin inhibits YAP/TAZ activity by disrupting the formation of the YAP/TAZ-TEAD complex [30]. Human TM cells cultured with 30 ng/mL VEGF were subsequently treated with 1 μM verteporfin. Relative to vehicle-treated human TM cells, the levels of fibrotic proteins were significantly decreased in verteporfin-treated cells (Figure 6). Furthermore, treatment with verteporfin markedly removed CLANs that were already formed. As illustrated in Figure 7, exposure to 30 ng/mL VEGF increased the percentage of CLAN-positive cells from 1.25% ± 0.43% to 35.13% ± 5.66% (*p* < 0.001), and subsequent treatment with verteporfin resolved CLAN structures, resulting in 5.32% ± 2.20% CLAN-positive cells (*p* < 0.001 vs. VEGF exposed cells). These observations suggest that TAZ might be crucial for high-VEGF-concentration-induced fibrosis and actin cytoskeleton changes in human TM cells.

## 3. Discussion

This study investigated the influence of low-to-high VEGF concentrations on human TM cells. We revealed that high VEGF concentrations significantly upregulate TAZ expression and nuclear translocation, increasing TAZ activity. Notably, increased TAZ activity was associated with upregulated fibrotic proteins, including fibronectin, collagen 1, and the CCN family proteins (CTGF and Cyr61), and accelerated CLAN formation in human TM cells. TAZ inhibition using verteporfin reversed the changes in the expression of fibrotic proteins and the actin cytoskeleton. Interestingly, exposure to a low VEGF concentration range elicited different responses in human TM cells, decreasing fibrotic protein expression. To the best of our knowledge, this is the first study to reveal the dose-dependent effects of VEGF on human TM cells. Given the importance of VEGF in ophthalmology, our findings are significant as they indicate that long-standing anti-VEGF therapy might impact the aqueous humor outflow facility. Additionally, they provide novel insights into TAZ as a novel therapeutic target for modulating outflow facilities in glaucoma.

VEGF regulates vasculogenesis and angiogenesis and has been studied extensively for its role in tumor pathogenesis, intraocular neovascular disorders, and other pathological conditions. However, the molecular mechanisms of how VEGF induces these effects have not been well defined. Recently, Wang et al. [24] linked the Hippo/YAP/TAZ signaling pathway and VEGF and suggested novel mechanistic insights into how VEGF controls the transcription of a subset of several genes in brain endothelial cells. They reported that YAP/TAZ activity is required in endothelial cells to build a transcriptional response that sustains VEGF signaling by controlling the expression of the gene subset that regulates the actin cytoskeleton. Similarly, Liu et al. observed that VEGF promotes TAZ expression, and this VEGF-mediated TAZ activation is essential for tumor angiogenesis [31,32]. Consistent with the prior results, we have observed that high VEGF concentrations activate TAZ in human TM cells. Our findings suggest that VEGF controls TAZ activity not only during angiogenesis in brain endothelial cells but also during fibrosis in human TM cells. Furthermore, these findings indicate that VEGF-driven responses in various pathologic ocular conditions might be mediated, at least partly, by TAZ signaling pathway activation.

YAP and TAZ are effective cell proliferation and survival regulators important in regulating critical cellular functions and normal tissue homeostasis [11,12]. Imbalance or failure of the YAP/TAZ signaling pathway are associated with multiple diseases, including atherosclerosis, fibrosis, cardiac hypertrophy, and cancer [33]. YAP/TAZ can be regulated by the F-actin cytoskeleton. They sense ECM stiffness and cell geometry, consequently affecting the cytoskeleton and cell mechanics via a feedback mechanism [11,12,13,14]. As mechanotransducers of the extracellular microenvironment, YAP/TAZ is upregulated by the elastic hardness of the human TM in glaucoma [15,16,17,18,19,20,21,22,23]. Since Raghunathan et al. [21] first demonstrated YAP/TAZ expression in human TM tissues, the pathological role of aberrant YAP/TAZ signaling in glaucoma has been an important topic of interest. YAP/TAZ nuclear localization, the principal mechanism to regulate their activity, was significantly higher in glaucomatous TM cells than in healthy TM cells. Moreover, Li et al. [20] demonstrated that inhibiting the YAP/TAZ signaling pathway ameliorates fibrotic protein deposition, subsequently decreasing ECM stiffness. Here, we have also uncovered that TAZ activation was associated with increased fibrosis and CLAN formation in human TM cells, confirming the results of previous studies.

Collagen is a major ECM component in the aqueous outflow pathway. Among the several collagen types, type 1 collagen occurs in the trabecular beam core—the basement membrane along the beams. They fulfil physical requirements given the biomechanical demands on the TM [34]. Fibronectin is another major ECM protein crucial in ECM organization, also acting as a bioreservoir for many proteins and growth factors essential in regulating aqueous humor outflow [34]. Meanwhile, CTGF and Cyr61 are the CCN family of matricellular proteins. Matricellular proteins do not play a structural role in supporting tissues but rather modulate the function and organization of the ECM as nonstructural secreted glycoproteins [4,35]. Overall turnover and balance between the structural and nonstructural ECM proteins contribute to the generation of a dysfunctional matrix in the human TM, affecting the outflow facility. Importantly, TGF-β2 promotes the expression of these ECM proteins. Thus, the pathological role of TGF-β2 in developing elevated IOP in glaucoma has been well established [36]. TGF-β2 induces nuclear YAP/TAZ localization and fibrosis-related target gene activation, leading to increased TM stiffness [19,37]. Previous findings that YAP- and TAZ-deficient fibroblasts were less reactive to TGF-β stimulation highlighted the close relationship between the TGF-β2 signaling pathway and YAP/TAZ activation [38]. In this study, we observed that exposure to high VEGF concentrations could accelerate fibrosis in a TAZ-mediated manner in human TM cells. Our findings suggest an additional mechanism whereby ECM changes, together with increased TGF-β expression, could contribute to an aqueous outflow facility.

Notably, there was a tendency for fibrotic protein decrease in human TM cells exposed to the low VEGF concentration (ρg/mL). Given that the VEGF concentrations in the aqueous humor in healthy individuals are approximately 20–100 ρg/mL, our findings suggest that VEGF might play a certain role in reducing outflow resistance at the TM level under physiological conditions [39,40,41,42,43]. The findings are consistent with previous reports regarding the association between VEGF and aqueous outflow [6,7,8,9]. VEGF’s role in the aqueous humor outflow facility has been documented in several studies [6,7,8,9]. Prior data have revealed that VEGF affects the aqueous humor outflow facility at the SC level by regulating endothelium permeability. VEGF decreases the barrier function of SC endothelium cells and increases the outflow facility in porcine anterior segments [7]. Moreover, pharmacologically blocking the VEGF receptor decreases the outflow facility in mice by suppressing endogenous VEGF signaling within the TM and SC. However, the mechanism by which the opposing responses are mediated via the YAP/TAZ signaling pathway is unknown. In the present study, we could not observe significant changes in the expression of TAZ and YAP and their nuclear localization at low VEGF concentrations. Further investigation might be needed to identify the physiological roles of VEGF in aqueous outflow and the underlying molecular mechanism. Nevertheless, based on these data, our findings highlight the importance of considering the possibility of disrupting IOP homeostasis by sustained exposure to anti-VEGF therapy.

Here, we did not observe significant changes in YAP protein and gene expression. In general, most of the molecular functions of YAP and TAZ overlap. However, several articles demonstrated that YAP and TAZ have distinct functions [25,44,45,46]. For instance, YAP and TAZ knockout mice exhibit different phenotypes. YAP null mice were not viable and lethal during embryogenesis. In contrast, TAZ null mice are viable but develop kidney and lung diseases [45,46]. The distinctive features between YAP and TAZ might be attributable to the differences in their structures, post-translational modifications, regulatory mechanisms, and interactors. Thus, these discrepancies activate transcriptional pathways that do not completely overlap. Our results further confirm the previous findings.

Confluent TM cells can undergo cytoskeletal rearrangements from linear stress fibers to form distinct geodesic dome-like structures of hubs and spokes known as CLANs. CLANs form more commonly in the glaucomatous TM cells and tissues than in the normal control cells and can also form in response to glucocorticoid or TGF-β2 treatments [27,28]. Predictive mathematical models suggested that CLAN formation is associated with IOP elevation by increasing the stiffness of actin filaments and overall cell stiffness [29]. In the present study, high VEGF concentrations induced CLAN formations in a TAZ-dependent manner. In addition, TAZ inhibition by verteporfin restored these structural alterations. Thus, a novel therapeutic strategy for targeting TAZ in VEGF-induced TM dysfunction might help lower IOP.

A high concentration of VEGF in aqueous humor is a major cause of neovascular glaucoma (NVG). Furthermore, recent studies have shown a trend towards elevated VEGF levels in the aqueous humor of patients with primary open angle glaucoma (POAG) [47]. Taken together, VEGF might play a certain role in increased aqueous outflow resistance in POAG as well as in NVG. From a clinical perspective, our findings suggest that TAZ inhibition might have a beneficial effect on the conventional outflow pathway by resolving structural changes in TM for glaucoma patients.

There are several limitations to this study. First, based on the results of the current study, the molecular mechanisms of the VEGF-induced increase in outflow facility at physiological conditions still remain unclear. Second, it is important to note that the findings presented here have been derived from cultured TM cells, which is a non-native environment. Further in vivo research might be necessary to determine whether our findings are applicable to animal models. Third, we focused on the structural changes of TM cells and did not determine their functional aspect. Whether structural changes are associated with functional abnormality in TM cells should be elucidated in future studies. Finally, we did not thoroughly evaluate the interaction between VEGF and its receptors (VEGFR) in human TM cells. The biological activities of VEGF are mainly mediated via two VEGFRs, VEGFR-1 and VEGFR-2 [8,48]. In the conventional outflow pathway, a signaling cascade through VEGFR-2 is reported to play a significant role [7,8,48]. Recently, neuropilin-1, which act as a co-receptor for VEGF, has been implicated in a variety of physiological and pathological process mediated by VEGF [49]. The role of specific VEGF receptors and their subsequent molecular pathways in human TM cells are the subject of our future study.

## 4. Materials and Methods

### 4.1. Human Trabecular Meshwork Cell Culture

Human TM cells from a normal human eye (Cat.# 6590) were purchased from ScienCell Research Laboratories (San Diego, CA, USA). The cells were cultured in trabecular meshwork cell media (TMCM; ScienCell) supplemented with 1% trabecular cell growth supplement, 2% fetal bovine serum (FBS), and 1% penicillin/streptomycin and maintained at 37 °C with 5% CO_2_ in a humidified atmosphere. Culture media were replaced every three days until 70% cell plate confluence. Then, the media were replaced daily, and cells were subcultured at 1:5 when they attained 90% confluence. Cells in passages 3–7 were used for all experiments. The TM cells were subjected to dexamethasone-induced myocilin expression as recommended to validate human TM cells (Supplementary Figure S1) [50]. Each experiment was repeated independently at least thrice. This was not deemed a human study because cells were acquired post-mortem from de-identified donor tissues and was exempted by the Chonnam National University Hospital Institutional Review Board (CNUH-EXP-2022-377).

### 4.2. Cell Viability

Cell viability was assessed using the Cell Counting Kit 8 ([CCK-8] Dojindo; Rockville, MD, USA) following the manufacturer’s protocol to evaluate VEGF cytotoxicity on human TM cells. Briefly, the cells were seeded at 5 × 10^3^ cells/well with complete culture media in a 96-well plate. The human TM cells were grown and subjected to recombinant human VEGF_165_ (R&D system; Minneapolis, MN, USA) over a concentration range (0, 1, 10, 30, 50, and 100 ng/mL). Among the several VEGF isoforms, VEGF_165_ was used in this study because it has been known to be expressed most abundantly in ocular tissue [48]. After 24 h, 10 μL of CCK-8 solution was added to each well, followed by incubation for 4 h at 37 °C. The absorbance was measured at 450 nm using a microplate reader. The mean absorbance from the eight wells was calculated for each VEGF concentration. Cell viability was expressed as a percentage of the control (untreated) cells.

### 4.3. Cell Treatments

**Figure 8:**
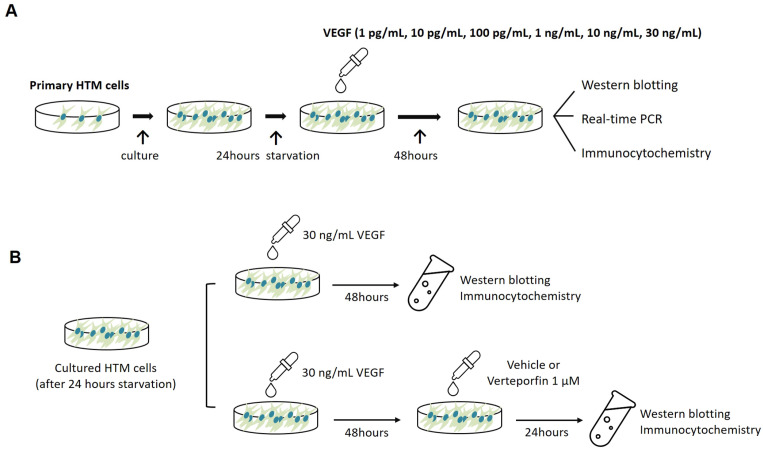
Schematic representation of experimental protocols in this study. Each experiment was repeated independently at least three times. (**A**) Human TM cells were subjected to different VEGF concentrations (1 ρg/mL, 10 ρg/mL, 100 ρg/mL, 1 ng/mL, 10 ng/mL, and 30 ng/mL). After 48 h, the cells were prepared for Western blotting, real-time PCR, or immunocytochemistry. (**B**) Human TM cells incubated with a high VEGF concentration (30 ng/mL) were divided into three groups based on the subsequent treatment protocol; VEGF only group: human TM cells were untreated after VEGF exposure; VEGF + Vehicle group: human TM cells were treated with vehicle solution for 24 h after VEGF exposure; VEGF + verteporfin group: human TM cells were treated with 1 μM verteporfin. After treatment, cells were prepared for Western blotting or immunocytochemistry.

Semiconfluent cultures of human TM cells were incubated overnight in serum-free media unless otherwise noted. Then, serum-starved cultures were subjected to different VEGF concentrations (1 ρg/mL, 10 ρg/mL, 100 ρg/mL, 1 ng/mL, 10 ng/mL, and 30 ng/mL) for 48 h (Figure 8A). In the control cultures, the medium was changed at the same time points, but without VEGF. In another set of experiments, the aforementioned experiments with 30 ng/mL VEGF were performed. The cells were divided into three groups based on the subsequent treatment protocol. (1) VEGF-only group: human TM cells were untreated after VEGF exposure; (2) VEGF + Vehicle group: human TM cells were treated with the vehicle (phosphate-buffered saline; PBS) solution for 24 h after exposure to VEGF; (3) VEGF + verteporfin group: human TM cells were treated with 1 μM verteporfin (Sigma-Aldrich; St. Louis, MO, USA) for 24 h after exposure to VEGF. In the control group, the medium was changed at the same time points without VEGF, vehicle, or verteporfin exposure. The verteporfin concentration used in this study has been verified as safe and efficacious in human TM cells and other ocular cells [15,51]. After the treatments, the cells were processed for Western blotting, quantitative real-time polymerase chain reaction (PCR), and immunocytochemistry (Figure 8B).

### 4.4. Quantitative Real-Time Polymerase Chain Reaction

The mRNAs of genes encoding YAP were evaluated using real-time PCR. Human TM cells were lysed and mRNA was extracted using TRIzol Reagent (Invitrogen, Grand Island, NY, USA). cDNA was synthesized using a PrimeScript 1st strand cDNA synthesis kit (Takara, Shiga, Japan) and analyzed via real-time PCR using a TB green Premix Ex Taq (Takara, Shiga, Japan) and a Thermal cycler Dice III (Takara, Shiga, Japan). Quantitative real-time PCR was performed on 10 ρmol/μL of cDNA with specific YAP primers. The primers used in the analysis were: forward 5′-ACCCACAGCTCAGCATCTTCG-3′ and reverse 5′-TGGCTTGTTCCCATCCATCAG-3′. All data were normalized to 18S-rRNA expression.

### 4.5. Western Blot

Human TM cells were lysed using RIPA lysis buffer (Biosesang, Seongnam, Republic of Korea). The protein lysates were centrifuged at 13,000 rpm for 15 min. The supernatants were collected and quantified using the Bradford protein assay method with protein assay dye reagent (Bio-Rad Laboratories, Hercule, CA, USA). Each sample (1 μg/mL) was separated onto 8% and 10% Tris-glycine gels. After protein transfer, membranes were blocked for 1 h at room temperature in tris-buffered saline–Tween-20 solution [TBS-T; 10 mmol/L Tris-HCl (pH 7.6), 150 mmol/L NaCl, and 0.1% Tween-20] containing 5% non-fat dry milk. After blocking, membranes were incubated overnight at 4 °C with a primary antibody recognizing the following proteins: TAZ (Cat.# 8418s, 1:1000; Cell Signaling Technology, Danvers, MA, USA), pTAZ (Ser89) (Cat.# AF4314, 1:1000; Affinity Biosciences, Cincinnati, OH, USA), YAP (Cat.# 14074S, 1:1000; Cell Signaling Technology), pYAP (Ser127) (Cat.# 13008S, 1:1000; Cell Signaling Technology), fibronectin (Cat.# NBP1-91258, 1:1000; NOVUS, Centennial, CO, USA), connective tissue growth factor (CTGF; Cat.# 86641S, 1:1000; Cell Signaling Technology), collagen 1 (Cat.# ab138492, 1:3,000; Abcam, Cambridge, UK), cysteine-rich angiogenic inducer 61 (Cyr61; Cat.# SC-374129, 1:1000; Santacruz biotechnology, Dallas, TX, USA), and β-actin. After three washes with TBS-T, the membranes were incubated for 1 h at room temperature with a peroxidase-conjugated goat anti-rabbit secondary antibody (Cat.# 7074S, 1:3000; Cell Signaling Technology) and anti-mouse IgG HRP-linked antibody (Cat.# 7076S, 1:3000; Cell Signaling Technology). Signals were visualized using an enhanced chemiluminescence HRP substrate (Minipore, Bedford, MA, USA) and quantified using an UVITEC Alliance Mini HD9 (UVITEC Ltd., Cambridge, UK).

### 4.6. Immunocytochemistry

Post-treatment, human TM cells were fixed with 4% paraformaldehyde (Biosesang, Seongnam, Republic of Korea) at room temperature for 15 min, permeabilized with 0.1% Triton X-100 (Thermo Fisher Scientific, Eugene, OR, USA) in PBS for 15 min at room temperature, and further blocked with blocking buffer using 1% BSA in PBS. Subsequently, coverslips were incubated overnight at 4 °C with a primary antibody against TAZ (1:100; Cell Signaling Technology). This was followed by incubation with a 1:200 dilution of an AlexaFluor 488-conjugated goat anti-rabbit IgG secondary antibody (Invitrogen) for 4 h at room temperature. Nuclei were counterstained with 4′,6-diamidino-2-phenylindole (DAPI; VECTOR, Burlingame, CA, USA). The fluorescent images were acquired using confocal microscopy on a laser scanning microscope (LSM 800; Carl Zeiss Microscopy, Peabody, MA, USA). For each immunolabelled glass coverslip, five random locations were imaged. At least five glass coverslips were used to identify each immunolabeling condition for each group with the same imaging setting.

### 4.7. Cross-Linked Actin Network Evaluation

Treated TM cells were fixed, permeabilized, and blocked using the method described above. F-actin was stained with phalloidin conjugated with AlexaFluor 488 (1:400; Invitrogen) for 1 h at room temperature. After PBS washes, coverslips were mounted onto slides using VECTASHIELD Antifade mounting medium with DAPI (VECTOR) for nuclear counterstaining. Cross-linked actin networks (CLANs) were visualized using the laser scanning microscope (LSM 800; Carl Zeiss Microscopy, Peabody, MA, USA). CLANs were defined as F-actin-containing cytoskeletal structures: at least one triangulated actin arrangement consisting of actin spokes and at least three identifiable hubs [27,37]. Each coverslip was assessed at five locations. At least five coverslips were evaluated per treatment group. To quantify the number of CLAN-positive cells, low-power (200×) fluorescence images were captured. The number of CLAN-positive cells per image and the total number of cells were counted to determine the percentage of CLAN-positive cells per image.

### 4.8. Statistical Analysis

Analyses were conducted on the SPSS software version 27.0 for Windows (SPSS Inc., Chicago, IL, USA). Data were expressed as mean ± standard error of the mean. One-way ANOVA with Turkey’s multiple comparisons post-hoc test was used to compare experimental groups. A *p*-value of < 0.05 was considered significant.

## 5. Conclusions

In summary, our findings may provide novel insights regarding the dose-dependent role of VEGF. We have revealed that exposure to high VEGF concentrations upregulated TAZ expression and drove TAZ nuclear localization in human TM cells, further activating the downstream signaling pathway associated with TM fibrotic change and CLAN formation. Moreover, TAZ inhibition by verteporfin reduced the expression of ECM proteins that promote TM fibrosis and restored cytoskeletal changes caused by high VEGF concentrations. In contrast, low VEGF concentrations decreased fibrotic protein expression in human TM cells. The role of VEGF under physiological conditions in the aqueous outflow pathway requires further elucidation. Nevertheless, our results reveal that patients can benefit from TAZ inhibition after developing pathological changes in TM induced by high VEGF concentrations. Additionally, TAZ may be a promising therapeutic target in future glaucoma treatment.

## Data Availability

Data are available upon reasonable request.

## Supplementary Materials

The following supporting information can be downloaded at: https://www.mdpi.com/article/10.3390/ijms24119625/s1.
