# Supplementary material for: High VEGF Concentrations Accelerate Human Trabecular Meshwork Fibrosis in a TAZ-Dependent Manner
Source: Int J Mol Sci. 2023 Jun 1;24(11):9625. doi: 10.3390/ijms24119625 (PMC10253844; doi:10.3390/ijms24119625)

Supplementary Material

High VEGF concentration accelerates human trabecular meshwork fibrosis in a TAZ-dependent manner

Mi Sun Sung,[1] So Young Kim,[1] Gwang Hyeon Eom,[2] and Sang Woo Park[1,*]

[1]Department of Ophthalmology, Chonnam National University Medical School and Hospital, Gwangju 61469, Republic of Korea

[2]Department of Pharmacology, Chonnam National University Medical School, Hwasun 58128, Republic of Korea

* Correspondence : exo70@naver.com

Figure S1: Validation of TM cell strain.

Primary human TM cells were cultured in the presence of 100nM dexamethasone (Dex) for 3 days. Immunoblot analyses showed that TM cells upregulate myocilin in response to dexamethasone treatment. *** $P < 0.001$ (Student's t-test)

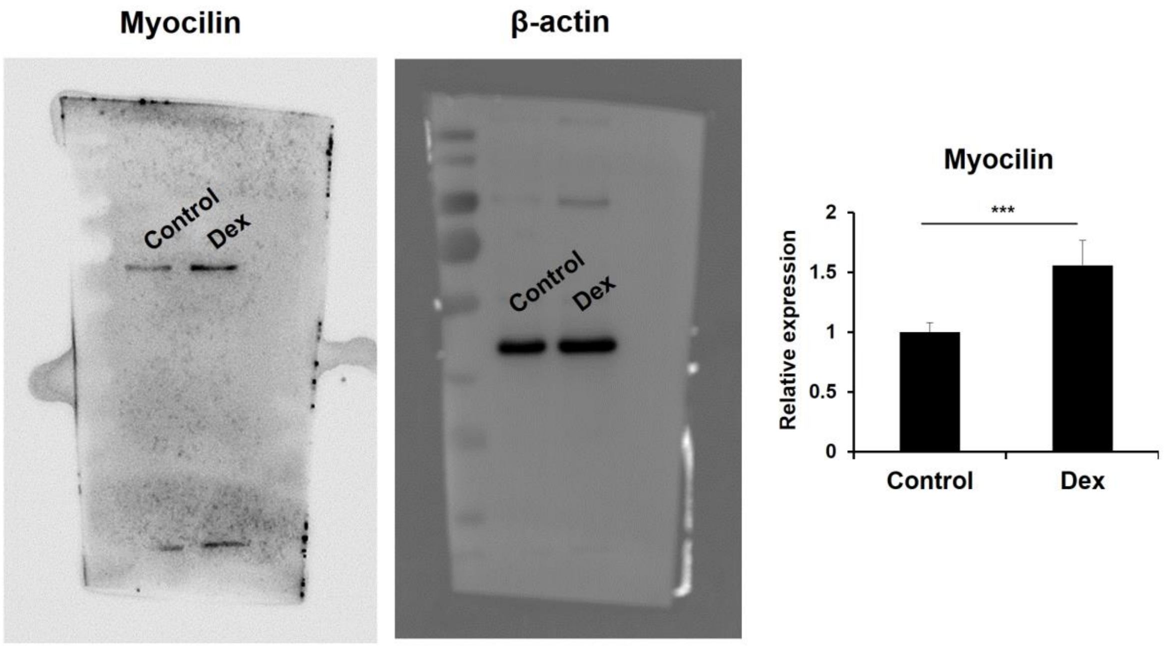

Supplement: Supplementary file 1 [file ijms-24-09625-s001.zip › ijms-2433324-supplementary.pdf]
